# Low birth weight and its associated risk factors in a rural health district of Burkina Faso: a cross sectional study

**DOI:** 10.1186/s12884-022-04554-w

**Published:** 2022-03-21

**Authors:** Moussa Lingani, Serge Henri Zango, Innocent Valéa, Georges Somé, Maïmouna Sanou, Sékou O. Samadoulougou, Serge Ouoba, Eli Rouamba, Annie Robert, Michèle Dramaix, Philippe Donnen, Halidou Tinto

**Affiliations:** 1 Institut de Recherche en Sciences de la Santé/Direction Régionale du Centre Ouest (IRSS/DRCO), Nanoro, Burkina Faso; 2 École de Santé publique, Université Libre de Bruxelles, Bruxelles, Belgium; 3 Epidemiology and Biostatistics Research Division, Institut de recherche expérimentale et clinique, Université catholique de Louvain, Bruxelles, Belgium; 4 Evaluation Platform on Obesity Prevention, Quebec Heart and Lung Institute Research Center, Quebec, Canada

**Keywords:** Low birth weight, Associated factors, Rural area, Burkina Faso

## Abstract

**Background:**

Low birth weight (LBW) is a major factor of neonate mortality that particularly affects developing countries. However, the scarcity of data to support decision making to reduce LBW occurrence is a major obstacle in sub-Saharan Africa. The aim of this research was to determine the prevalence and associated factors of LBW at the Yako health district in a rural area of Burkina Faso.

**Methods:**

A cross sectional survey was conducted at four peripheral health centers among mothers and their newly delivered babies. The mothers’ socio-demographic and obstetrical characteristics were collected by face-to-face interview or by review of antenatal care books. Maternal malaria was tested by standard microscopy and neonates’ birth weights were documented. Multivariate logistic regression was used to determine factors associated with LBW. A *p*-value < 0.05 was considered statistically significant.

**Results:**

Of 600 neonates examined, the prevalence of low birth weight was 11.0%. Adjustment for socio-demographic characteristic, medical conditions, obstetrical history, malaria prevention measures by multivariate logistic regression found that being a primigravid mother (aOR = 1.8, [95% CI: 1.1–3.0]), the presence of malaria infection (aOR = 1.9, [95% CI: 1.1–3.5]), the uptake of less than three doses of sulfadoxine-pyrimethamine for the intermittent preventive treatment of malaria in pregnancy (IPTp-SP) (aOR = 2.2, [95% CI: 1.3–3.9]), the presence of maternal fever at the time of delivery (aOR = 2.8, [95% CI: 1.5–5.3]) and being a female neonate (aOR = 1.9, [95% CI: 1.1–3.3]) were independently associated with an increased risk of LBW occurrence. The number of antenatal visits performed by the mother during her pregnancy did not provide any direct protection for low birth weight.

**Conclusion:**

The prevalence of LBW remained high in the study area. Maternal malaria, fever and low uptake of sulfadoxine-pyrimethamine doses were significantly associated with LBW and should be adequately addressed by public health interventions.

## Introduction

Low birth weight (LBW) is a leading determinant of neonatal mortality worldwide that particularly affects the developing countries [1]. In the Sub-Saharan African (SSA) regions, 5 million low birth weight babies were recorded in 2015, of those 20% were secondary to malaria in pregnancy [2, 3] and the other causes included the maternal socio-demographic, gyneco-obstetrical characteristics and the fetus genetic disorders [4, 5]. Despite that LBW association with neonatal death is well established in SSA, half of all birth weights is still not recorded due weaknesses in the routine systems, which understates the magnitude of the problem and delays decision making [1, 6]. In some selected countries, the reported prevalence varied between 8 and 17% [7]. In Burkina Faso, the prevalence was 15.8% in 2005 and decreased to 13.4% in 2011 [7, 8].

To reduced malaria related adverse birth outcomes, the intermittent preventive treatment of malaria in pregnancy using sulfadoxine-pyrimethamine (IPTp-SP) in combination with vector control approaches such as free distribution of insecticide treated bed nets (ITNs) were adopted [9]. From an initial two-doses policy in the 2000s, the approach was revised in 2012, and a minimum of three doses of SP were recommended [9, 10]. The strategy consists of the administration of a full treatment course of sulfadoxine-pyrimethamine (SP) to pregnant women from the second trimester, monthly until delivery [9], and was proven efficacious [11]. The increasing trends of *P. falciparum* resistance to SP, particularly in western and central African regions are however raising concerns regarding the efficacy of the strategy [12]. Indeed, high prevalence of triple and quadruple mutations in the dihydropteroate synthase *(dhps)* and dihydrofolate reductase *(dhfr*) genes of *P. falciparum* are widely reported [13]. Also, the magnitude of the quintuple and sextuple mutations in the *dhps* and *dhfr* genes along with the emergence of septuple even octuple mutations reported in several countries is a further threat for the efficacy of the strategy and is reported to cause low birth weight in sub-Saharan Africa [14–17].

Efforts were deployed in different countries to mitigate the effects of the SP resistance through a supervision of the administration of the drug in pregnant women and also by increasing the number of SP doses required to a minimum of three during a pregnancy course. In 2013, Burkina Faso adopted the new policy [18, 19] and given the risk of decreasing efficacy, a continuous assessment of the strategy’s efficacy on the reduction of adverse birth outcomes is needed. However, since the adoption of the new policy, no published data estimated the magnitude of low birth weight in the country [20] despite the growing concerns regarding *P. falciparum* resistance to SP [12]. The purpose of this study was to measure the prevalence of low birth weight and to determine its associated factors in a rural area of Burkina Faso 5 years after the recommended three-doses IPTp-SP policy was adopted.

## Methods

### Study design and settings

**Fig. 1:**
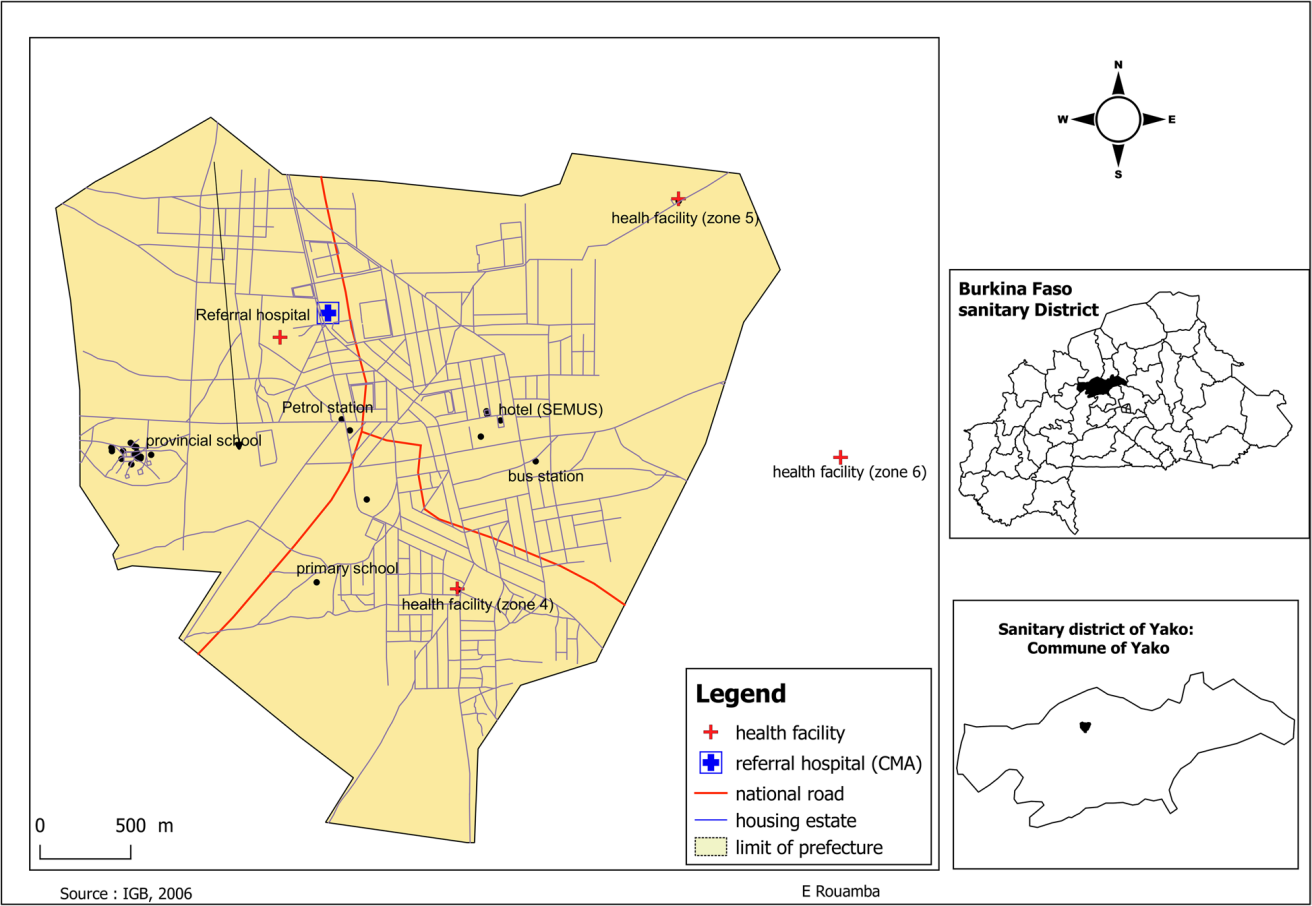
Map of the Yako commune with the four recruitment sites, Burkina Faso

A cross-sectional survey was conducted at four health centers of the Yako health district from August 2019 to March 2020 (Fig. 1). In Burkina Faso, malaria transmission is holo-endemic with a marked seasonal transmission that overlaps with the rainy season (July–November) [21]. The Yako health district covered a total of 424,577 inhabitants in 2017, and 22,500 pregnancies were recorded [21].

### Sampling procedures

All nursing mothers who delivered a single live neonate within the past 24 h were enrolled into the study. These mothers were systematically recruited on a daily basis in a sequential manner as they visited the selected health centers for care until completion of the required sample size.

### Participants’ eligibility criteria

Mothers were included if they were aged between 15 and 45 years, had a gestational age greater or equal to 37 weeks, lived in the study catchment area and provided a written informed consent. They were not included if they had twin pregnancies or any condition known to interfere with birth outcome. Participants were subsequently excluded if the neonate could not be examined within 24 h of delivery due to neonate death or conditions that required reference to a larger hospital prior to examination by the study staff.

### Sample size estimation

At the time of the IPTp-SP policy changed, the prevalence of low birth weight in Burkina Faso was 13.4% [7]. We hypothesized that the new policy would reduce the prevalence to 10% within 5 years. The required sample size was calculated using the Cochran formula *n* = Z^2^ *p*(1-p)/i^2^ where *p* = 10% was the expected proportion, i = 2.5%, the margin of error, and Z the z-score that corresponds to the 95% confidence interval (1.96), The minimum sample size was 554 participants.

### Data collection procedures and main variables’ definitions

Data was extracted from the mothers’ ANC books or obtained by interview of the mothers when the information was not available in the ANC books and subsequently recorded onto semi-structured questionnaires by trained midwives. Age, gyneco-obstetrical history, IPTp-SP uptake, educational level, occupation, and the use of bed nets the night before admission for delivery were collected. In addition to physical and obstetrical examinations, blood pressure, axillary temperature, and body weight were measured, completed by malaria diagnosis in peripheral blood samples. Due to the absence of ultrasounds, we estimated gestational age using the knowledge of the last menstrual period (LMP), or the Ballard score whenever the LMP was unknown. Neonates’ birth weights were measured with calibrated Seca® 384 electronic scales with 10 g resolution and a precision of 5 g (Seca gmbh & co. kg, Germany).

The outcome variable was birth weight measured within 24 h of delivery, and was dichotomized in low (< 2500 g) and normal (≥ 2500 g) birth weights [22]. Explanatory variables included malaria infection (any density of asexual malaria parasite or positive rapid diagnosis test), IPTp-SP uptake (optimal if at least three doses, and non-optimal if less than 3 doses), number of pregnancies (1, 2–4 or ≥ 5), body mass index (BMI) (body weight in kilograms) divided by the square of the height (in meters) (< 18.5 and ≥ 18.5), history of miscarriage or stillbirth, high blood pressure (systolic pressure ≥ 140 mmHg and/or diastolic pressure ≥ 90 mmHg), fever (axillary temperature ≥ 37.5 degree centigrade), level of education (none, primary, secondary or plus), occupation (unemployed, employed/self-employed) and sleeping under a bed net the night before hospital admission (Table 1).

**Table 1:** List and definition of main variables

	Variables	Definitions	Categories
1	Age	Mother age in years	< 20, 20–34, ≥ 35
2	Educational level	Educational level	none, primary, secondary - plus
3	Occupation	Mother’s occupation	unemployed, employed/self employed
4	Gravidity	Number of pregnancies	primigravidae (1), paucigravidae(2–4), and multigravidae (≥ 5)
5	Miscarriage/stillbirth	Maternal history of stillbirth or abortion	yes / no
6	Body weight	The mother body weight	in kilograms
7	Height	Mother height	in meters
8	BMI (in kg/m^2^)	Maternal body mass index (BMI): ratio of maternal weight (in kilograms) over the square of maternal height (in meters) (in kg/m^2^)	low (< 18.5), normal or high (≥ 18.5)
9	Blood pressure	Maternal blood pressure	in mmHg
10	HBP	High blood pressure: systolic ≥140 and/or diastolic ≥90	yes / no
11	ITN use	Use of ITN the night before admission	yes / no
12	IPTp-SP	Number of IPTp - SP doses received	non-optimal (< 3), optimal (≥ 3)
13	Fever	Fever in the last 24 h or temperature > =37.5	yes / no
14	Malaria infection	Positive blood smear or positive rapid test	no / yes
15	Sex of newborn	Sex of the neonate	male, female
16	Birth weight	Neonate birth weight	in grams

*ANC* antenatal care, *ITN* Insecticide-treated bed net, *IPTp-SP* intermittent preventive treatment of malaria in pregnancy using sulfadoxine-pyrimethamine, *HBP* high blood pressure

### Laboratory procedures

Thick and thin blood smears were prepared, dried and stained with 5% Giemsa for 30 min. Two independent microscopists examined the slides at 1000x magnification using light microscopy. Parasite densities were calculated by counting the number of asexual parasites per 200 white blood cells (WBC), and parasites per μl calculated assuming WBC count of 8000 cells per μl of whole blood. When the number of asexual parasites was less than 100 per 200 WBC, counting was done against at least 500 WBC. A slide was considered negative if no parasite was found after a review of 1000 WBC or 100 fields containing at least 10 WBC per field. In case of discrepant results (discrepant species or count difference of at least 50% between the two microscopists), a third microscopist assessed the slides and the average of the two closest reads was used.

### Data processing and analysis

Data were collected on electronic questionnaires using REDcap (Research Electronic Data Capture) data collection tool and exported onto Stata version 15 (StataCorp. 2017, TX, USA) for cleaning and analysis. Categorical variables were summarized on frequency tables. Mean or median with respective standard deviations or quartiles were used to summarize numerical variables. T-test was used to compare means and odds ratios (OR) with 95% confidence intervals (95% CI) calculated by univariable logistic regression. Adjusted OR (aOR) were derived by backward multivariable logistic regression of factors which *p*-values were < 0.1 at univariable analysis and keeping those with *p*-values < 0.1 in the final model. Maternal age was not included in the model because of its strong correlation with the number of pregnancies. In addition, gravidity was dichotomized (1 or ≥ 2 deliveries) for birth weight factors assessment. The significance level was set at 5% (two-sided *p*-value).

## Results

### Study participants background characteristics

A total of 684 participants were assessed, 600 (87.7%) were included and 84 (12.3%) not eligible. Reasons for non-eligibility included 12 preterm births, 18 non-singleton births, 12 stillbirths, 2 miscarriages, 5 neonatal deaths, 15 data losses due to electronic pad breakdown, 16 concurrent participations to another research and 4 very ill neonates referred with missing birth weights.

Majority of women were unemployed (69.2%) with none or primary level of education (67.8%). Mean maternal age was 25 ± 6 (range; 15–42) years and 23% were aged less than 20 years. The median number of pregnancies stood at two (interquartile range, IQR; 1–4). Regarding malaria prevention measures, 92% stated using ITN the night before admission to delivery clinic and the IPTp-SP uptake was 77, 17.0, 5.0 and 1% for 3 or more doses, 2 doses, one dose and no dose respectively. Malaria infection was diagnosed in 17.5% (105/600) of participants, among them 83.8% were asymptomatic (Table 2).

**Table 2:** Socio-demographic, gyneco-obstetric and medical characteristics of study participants in Yako health district, Burkina Faso (*n* = 600)

Characteristics	Items	Total	Percentage
Age (years)	< 20	138	23.0
	20–35	407	67.8
	≥ 35	55	9.2
Educational level	None,	320	53.4
	Primary	86	14.4
	Secondary- plus	193	32.2
Occupation	Unemployed	413	69.2
	Employed/self employed	184	30.8
Gravidity	Primigravidae	198	33.0
	Paucigravidae	306	51.1
	Multigravidae	95	15.9
History of miscarriage / stillbirth	No	537	91.8
	Yes	48	8.2
BMI (in kg/m^2^)	< 18.5	28	4.7
	≥ 18.5	565	95.3
HBP	No	577	96.2
	Yes	23	3.8
ITN use	No	42	7.1
	Yes	550	92.9
IPTp-SP (doses)	< 3	138	23.0
	≥ 3	461	77.0
Fever	No	525	87.5
	Yes	75	12.5
Malaria infection	No	495	82.5
	Yes	105	17.5
Sex of newborn	Female	310	51.7
	Male	290	48.3

Abbreviations. *SP* sulfadoxine-pyrimethamine, *IPTp* intermittent preventive treatment of malaria in pregnancy, *CI* confidence interval, *HBP* high blood pressure, *ITN* Insecticide treated bed net, *BMI* body mass index

### Factors associated with neonate low birth weight

Among the 600 neonates examined 51.7% were females. The prevalence of low birth weight was 11%, and the mean birth weight was 2942 ± 459 g. Mean birth weight was significantly lower in female neonates compare to males (2893.5 versus 2993.9 g, *p* < 0.01), in neonates from mothers with less than three SP doses than those with three or more doses (2821.1 versus 2978.7 g, *p* < 0.001), and if the mothers had malaria infection (2858.7 versus 2959.7 g, *p* = 0.02). By multivariate analysis, first pregnancy, malaria infection, uptake of less than three SP doses, maternal fever and being a female neonate were independently associated with an increased risk of LBW (Table 3).

**Table 3:** Prevalence of low birth weight and associated factors in rural Burkina Faso 2019–20 (*n* = 600)

Characteristics	N	LWB (%)	OR [95%CI]	*p*-value	^1^aOR [95%CI]	*p*-value
**Overall**	600	11.0	–	–	–	–
**Educational level**				0.889		
None	320	10.6	0.9 [0.5–1.5]		–	
Primary	86	10.5	0.9 [0.4–1.9]			
Secondary - plus	193	11.9	Ref			
**Occupation**				0.337		
Unemployed	413	11.9	1.3 [0.7–2.4]		–	
Employed/self employed	184	9.2	Ref			
**Parity**				0.013		0.039
1	198	15.7	1.9 [1.2–3.2] *		1.8 [1.1–3.0] *	
2 or more	401	2.1	Ref		Ref	
**History of miscarriage/ stillbirth**				0.216		
Yes	48	6.3	0.5 [0.2–1.7]		–	
No	537	11.7	Ref			
**BMI (kg/m**^**2**^**)**				0.265		
< 18.5	65	17.9	1.8 [0.7–4.9]		–	
≥ 18.5	528	10.6	Ref			
**HBP**				0.130		
Yes	23	21.7	2.3 [0.8–6.5]		–	
No	577	10.6	Ref			
**ITN use**				0.776		
No	42	9.5	0.9 [0.3–2.5]		–	
Yes	550	10.9	Ref			
**IPTp-SP (doses)**				0.003		0.004
< 3	138	18.1	2.3 [1.3–3.9] **		2.3 [1.3–3.9] **	
≥ 3	461	8.9	Ref		Ref	
**Fever**				0.001		0.001
Yes	75	24.0	3.1 [1.7–5.8] ***		2.8 [1.5–5.3] **	
No	525	9.1	Ref		Ref	
**Malaria infection**				0.016		0.034
Yes	105	18.1	2.1 [1.2–3.8] *		1.9 [1.1–3.5] *	
No	495	9.5	Ref		Ref	
**Sex of newborn**				0.019		0.023
Female	310	13.9	1.9 [1.1–3.2] *		1.9 [1.1–3.3] *	
Male	290	7.9	Ref		Ref	

Abbreviations. *SP* sulfadoxine-pyrimethamine, *IPTp* intermittent preventive treatment of malaria in pregnancy, *CI* confidence interval, *LBW* low birth weight, *HBP* high blood pressure, *Ref* reference group, *ITN* Insecticide-treated bed net, *HBP* high blood pressure, *kg/m*^*2*^ kilogram per square meter

^1^Variables gravidity, IPTp-SP, fever, malaria infection and sex of newborn were included in the multivariate analysis; * *p* < 0.05, ** *p* < 0.01, *** *p* < 0.001

## Discussion

This paper examined the prevalence and associated factors of low birth weight in rural health centers in Burkina Faso 5 years after the start of the three doses IPTp-SP policy. Importantly, the study was conducted in the rural area where the prevalence of low birth weight secondary to malaria was the highest [23]. To conduct this study, we hypothesized that low birth weight prevalence would be reduced from 13.4 to 10% 5 years after the IPTp-SP policy change [7]. In this study, we reported a prevalence higher than that expected (11.0%) and this advocates that more efficacious interventions are needed to reduce LBW prevalence. In the literature, low neonate birth weight is usually related to situations where fetal intrauterine malnutrition is produced due to alterations in the placental blood flow which originates from several factors including the mother socio-economic status, inadequate pregnancy care, or infections [24].

We found that the uptake of less than three doses of SP was significantly associated with a higher prevalence of low birth weight and a significant lower mean birth weight. This finding supports that the new IPTp-SP policy remains efficacious in the study settings to reduce low birth weight prevalence, and advocates more efforts to reduced non optimal uptake of SP. The reason behind the efficacy of the new policy could be related to the reduction of the placental inflammation caused by infectious pathogens [25], which reduces the dysregulation of the placental angiogenesis secondary to these infections, source of intrauterine growth retardation [26]. Also, the number of SP doses administered to women during pregnancy could be considered as a surrogate of the quality of care provided to pregnant women. Indeed, as the anti-malarial is administered under health care workers’ supervision, each contact represents an opportunity to detect and address maternal conditions that may affect the birth weight at delivery. Thus, increasing the number of SP doses, increases the number of contacts with the health care workers and improve the quality of care.

As expected [3], malaria was associated with an increased risk of LBW in the study area. This indicates that despite widespread use of malaria prevention methods, it remains a major factor of low birth weight in the rural area of Burkina Faso. *Plasmodium* resistance to sulfadoxine pyrimethamine reported in sub-Saharan Africa and Burkina Faso, could be the main reason of this reduced efficacy [14–17]. Indeed, the high number of triple and the presence of quintuple mutations (triple *dhfr* and double *dhps* mutations) reported in Burkina Faso decreased the efficacy of this antimalarial to reduce adverse birth outcomes [27]. Therefore, as well as the need to update resistance level grows [27, 28], it becomes also necessary to assess alternative interventions. Mefloquine and azithromycin-based combinations were tested and are considered equivalent to the current approach although more evidence is needed [29].

We noted that first pregnancy infants were at particular higher risk of LBW [30–32]. Several factors including the physiological [33–35], structural and immunological [36] patterns of the uterus could impact the placental blood flow and induced inadequate fetal growth. In addition, the higher predisposition of first pregnancy placenta to malaria parasites’ sequestration put the first pregnancy fetus at higher risk of LBW [37]. A special attention should be paid to those women, and tight supervision of these pregnancies are required in the rural area.

As, already reported [38], female neonates were at higher risk of low birth weight. The difficulty in this study was that neonate sex was determined only after delivery which limits the utility of the information for decision making. However, in settings where ultrasounds are available, it would be useful to tailor pregnancy care to the fetus sex.

In this study we described the prevalence of low birth weight and tentatively identified some risk factors in women from Yako health district. However, some limitations are worth noting. The consideration of term pregnancy neonates and the exclusion of twin deliveries and stillbirths somehow understate the actual magnitude of low birth weight at the site. Gestational age was collected using the mother knowledge of last menstrual period or the Ballard score due to the absence of ultrasounds, and this is frequently prone to errors. Also, the cross-sectional design of the study does not guaranty causality between exposures and outcomes. However, the study is worth underlying the extent of some factors influencing adequate maternal and child health and help identify areas with the most need of interventions.

## Conclusion

Low birth weight prevalence was high in the study area. Reducing the proportion of pregnant women with low IPTp-SP uptake and malaria infection could reduce low birth weight occurrence and improve neonatal survival particularly among infants born from young mothers.

## Data Availability

The dataset analyzed for this manuscript is available at https://datadryad.org/stash/share/HWpwCD-7w_wTW_ALhVlIC_7OJNZU9eH2poq68kgvxe4.

## Abbreviations

MiP: Malaria in pregnancy
SP: Sulfadoxine-pyrimethamine
IPTp: Intermittent preventive treatment in pregnancy
LBW: Low birth weight
ANC: Antenatal care
OR: Odds ratio
aOR: Adjusted odds ratio
CI: Confident interval
WHO: World health organization
LMP: Last menstrual period
CRUN: Clinical Research Unit of Nanoro
